# Plastomes of nine hornbeams and phylogenetic implications

**DOI:** 10.1002/ece3.4414

**Published:** 2018-08-07

**Authors:** Ying Li, Yongzhi Yang, Le Yu, Xin Du, Guangpeng Ren

**Affiliations:** ^1^ State Key Laboratory of Grassland Agro‐Ecosystem School of Life Sciences Lanzhou University Lanzhou Gansu China

**Keywords:** *Carpinus*, coding genes, complete chloroplast genome, phylogenetics

## Abstract

Poor phylogenetic resolution and inconsistency of gene trees are major complications when attempting to construct trees of life for various groups of organisms. In this study, we addressed these issues in analyses of the genus *Carpinus* (hornbeams) of the Betulaceae. We assembled and annotated the chloroplast (cp) genomes (plastomes) of nine hornbeams representing main clades previously distinguished in this genus. All nine plastomes are highly conserved, with four regions, and about 158–160 kb long, including 121–123 genes. Phylogenetic analyses of whole plastome sequences, noncoding sequences, and the well‐aligned coding genes resulted in high resolution of the sampled species in contrast to the failure based on a few cpDNA markers. Phylogenetic relationships in a few clades based only on the coding genes are slightly inconsistent with those based on the noncoding and total plastome datasets. Moreover, these plastome trees are highly incongruent with those based on bi‐parentally inherited internal transcribed spacer (ITS) sequence variations. Such high inconsistencies suggest widespread occurrence of incomplete lineage sorting and hybrid introgression during diversification of these hornbeams.

## 1 INTRODUCTION

Poor phylogenetic resolution and inconsistency of gene trees are major complications when attempting to construct trees of life for various groups of organisms, as illustrated by numerous authors (e.g., Mallet, Besansky, & Hahn, 2016; Ren, Conti, & Salamin, 2015; Xu, Wu, Gao, & Zhang, 2012; Zeng et al., 2017; Zhang & Li, 2011). In this study, we addressed these issues in analyses of the genus *Carpinus* L. (hornbeams), subfamily Coryloideae, of the Betulaceae. The genus includes more than 40 recognized species (Grimm & Renner, 2013; Li & Skvortsov, 1999), most of which are distributed in China (Li & Skvortsov, 1999). The species of this genus are usually small trees or shrubs (Grimm & Renner, 2013), and most of them are used as important ornamental plants due to their lush foliage, graceful form, clustered fruits, and high stress resistance. In addition, chemicals extracted from this genus, including flavonoids and pheophorbides from leaves, have potential anti‐carcinogenic activities (Cieckiewicz, Angenot, Gras, Kiss, & Frederich, 2012; Sheng et al., 2016). Despite the importance of this genus, phylogenetic relationships of its major clades remain unresolved. Previous phylogenetic analyses based on three chloroplast (cp) DNA fragments (*mat*K, *trn*L‐*trn*F, and *psb*A‐*trn*H) and the nuclear internal transcribed spacer (ITS) region have identified four and five major clades in this genus, respectively. Moreover, resolutions of phylogenetic trees based on the two datasets are generally low, and the phylogenetic relationships they indicate are incongruent (Yoo & Wen, 2007). Similar problems have been encountered in phylogenetic analyses of the closely related genus *Ostrya* (Lu et al., 2016). The poor resolution of the cited cpDNA phylogeny was probably at least partly due to insufficient informative sites in the sampled sequences, and clearly full plastome sequencing would improve the resolution (e.g., Hu et al., 2015, 2016). Furthermore, well‐resolved cpDNA phylogenies would greatly facilitate clarification of reticulate evolution during species diversification (Jansen et al., 2007; Xu et al., 2012).

With the development of high‐throughput sequencing technology, it is becoming much cheaper and easier to sequence whole plastomes of plants (Hu et al., 2016; Zhang & Li, 2011) and thus increase the resolution of previously ambiguous phylogenetic relationships based on several cpDNA markers (Hu et al., 2016; Jansen et al., 2007). For example, Zeng et al. (2017) used whole plastome sequences and coding genes to construct phylogenetic trees of *Rehmannia*, both of which indicated four nearly identical clades and had high levels of phylogenetic resolution. The noncoding regions in a plastome usually have higher variation rates than the coding genes (Hu et al., 2016; Zhang & Li, 2011). However, it is not known whether phylogenetic trees based on noncoding sequences and coding genes of plastomes of the genus *Carpinus* would be consistent. Thus, in this study, we sequenced plastomes of nine species representing four major clades of the genus identified in a previous study (Yoo & Wen, 2007). We examined structural variations of the plastomes among the species, extracted three sets of sequences (whole plastomes, noncoding sequences, and coding genes), for phylogenetic analyses and compared the resulting trees with the ITS trees. We specifically addressed the following three questions. Does use of the three plastomic datasets covering more informative sites provide greater phylogenetic resolution of the sampled clades than use of a few cpDNA markers? Are phylogenies based on the three datasets consistent? Are phylogenies based on plastome datasets consistent with those based on nuclear ITS sequences?

## 2 MATERIALS AND METHODS

### 2.1 Plant materials, DNA extraction, and ITS sequencing

We chose nine species (i.e., *C. fangiana, C. cordata, C. betulus, C. caroliniana, C. fargesiana, C. tschonoskii, C. putoensis*,* C. tientaiensis,* and *C. viminea*) to represent the four clades based on ITS sequence variation (Yoo & Wen, 2007). According to Kuang and Li (1979), based on characters of bracts and nutlets, *C. fangiana* and *C. cordata* belong to section *Distegocarpus*, and the other seven species belong to section *Carpinus*. As *C. betulus* and *C. caroliniana* are distributed in Europe and North America, respectively, it was difficult for us to obtain fresh leaves of these species from the field. We therefore used a specimen of *C. betulus* collected in Dagestan in 1987 and a specimen of *C. caroliniana* collected in USA in 1996. Fresh leaves of the remaining seven species were collected in the field and dried immediately in the presence of silica gel (Table S1). We could not get any samples of the three species included in one of the ITS clades identified by Yoo and Wen (2007): *C. monbeigiana*,* C. pubescens,* and *C. turzaninowii*. However, our initial analysis of ITS sequences suggested that *C. fargesiana* is closely related to *C. turczaninowii* and thus could be used to represent this ITS clade. We selected *Corylus fargesii* as an outgroup. We used the modified CTAB method to extract total DNA from the dried leaves (Doyle & Doyle, 1987). ITS sequences of four *Carpinus* species (*C. betulus, C. caroliniana, C. putoensis,* and *C. tientaiensis*) and the outgroup species (*Corylus fargesii*) were downloaded from GenBank, while we sequenced samples from 5 to 10 individuals of each of the other species to obtain their ITS sequences (Table S2).

### 2.2 Plastome sequencing, assembly, and annotation

Following well‐established protocols (van Dijk, Jaszczyszyn, & Thermes, 2014), we prepared end‐repaired, phosphorylated and A‐tailed DNA fragments ligated with index adapters. We amplified the ligated fragments and constructed paired‐end libraries (2 × 150 bp), which we sequenced using a Hiseq Platform (Illumina, San Diego, CA). We filtered adapters from the sequence data and extracted high‐quality reads (MINLEN > 36, *Q* ≥ 5) using Trimmomatic v.0.32 (Bolger, Lohse, & Usadel, 2014) and the *Ostrya rehderiana* plastome as a reference (Li et al., 2016). We separated the plastome reads using Bowtie2 v.2.2.9 (Langmead & Salzberg, 2012) and utilized SAMtools v.1.3.1 (Li et al., 2009) to convert the SAM file to a BAM file. We then used bam2fastq v1.1.0 (Lindenbaum, 2015) to extract and map the short reads to the reference genome in order to generate a FASTQ file for subsequent plastome assembly by Velvet v.1.2.10 (Zerbino & Birney, 2008). We used BWA v.0.7.12 (Li & Durbin, 2009) to build an index and map all of plastome sequences to the reference plastome via the mem algorithm. The output files were converted and sorted using SAMtools v.1.3.1 (Li et al., 2009). We used Geneious v.10 (Kearse et al., 2012) to visualize the assembled results, and Plann v.1.1.2 (Huang & Cronk, 2015) to annotate plastomes and Sequin v.15.10 (http://www.ncbi.nlm.nih.gov/Sequin/) to map the predicted genes to the reference annotation. Visual images of the annotations were generated by OGDRAW v.1.1 (http://ogdraw.mpimp-golm.mpg.de/; Lohse et al. 2013). To graphically display interspecific variations, the alignments with annotations of nine plastomes were plotted using mVISTA (Mayor et al., 2000).

### 2.3 Phylogenetic analyses

We aligned the plastome and ITS sequences of the nine selected *Carpinus* species and the outgroup using MAFFT v.7 (Katoh, Misawa, Kuma, & Miyata, 2002) and MEGA v.6 (Tamura, Stecher, Peterson, Filipski, & Kumar, 2013). The aligned sequence matrix was then manually examined and corrected. To assess the consistency of phylogenetic constructions based on different plastome regions, we extracted three datasets from the finally aligned plastome matrix. These included sequences of: (a) the whole plastomes, (b) noncoding regions, and (c) protein‐coding genes (PCGs) present in all nine *Carpinus* species and the outgroup. We converted FASTA files to NEXUS or PHYLIP format using ClustalW v.2.1 (Larkin et al., 2007). All alignment positions containing gaps in one or more taxa were removed before phylogenetic analyses. We used Prank v. 6.864b (Loytynoja & Goldman, 2010) to align coding genes. We estimated constant sites, parsimony informative sites, and variable sites of the three plastome datasets and ITS matrix using MEGA v.6 (Tamura et al., 2013). For ITS sequences, we only retained one haplotype if multiple identical haplotypes existed within each species for the phylogenetic analyses. MrBayes v.3.2.4 (Huelsenbeck & Ronquist, 2001) was used to reconstruct phylogenetic trees. We repeated the MrBayes analyses three times for each of the datasets (i.e., the whole plastomes, noncoding regions, coding genes, and ITS sequences); in each case running four chains (one cold and three hot) of 10,000,000 generations, sampling every 1,000 steps with the temperature parameter set to 0.1. We determined convergence by examining trace plots of the log likelihood values for each parameter in Tracer v.1.6 (Rambaut, Xie, & Drummond, 2014). Maximum‐likelihood (ML) analyses were performed with RAxML v.8.1.17 (Stamatakis, 2014) using the GTR + G model of evolution and 1,000 bootstrap replicates to assess node support.

## 3 RESULTS

### 3.1 Plastome features

**Figure 1:**
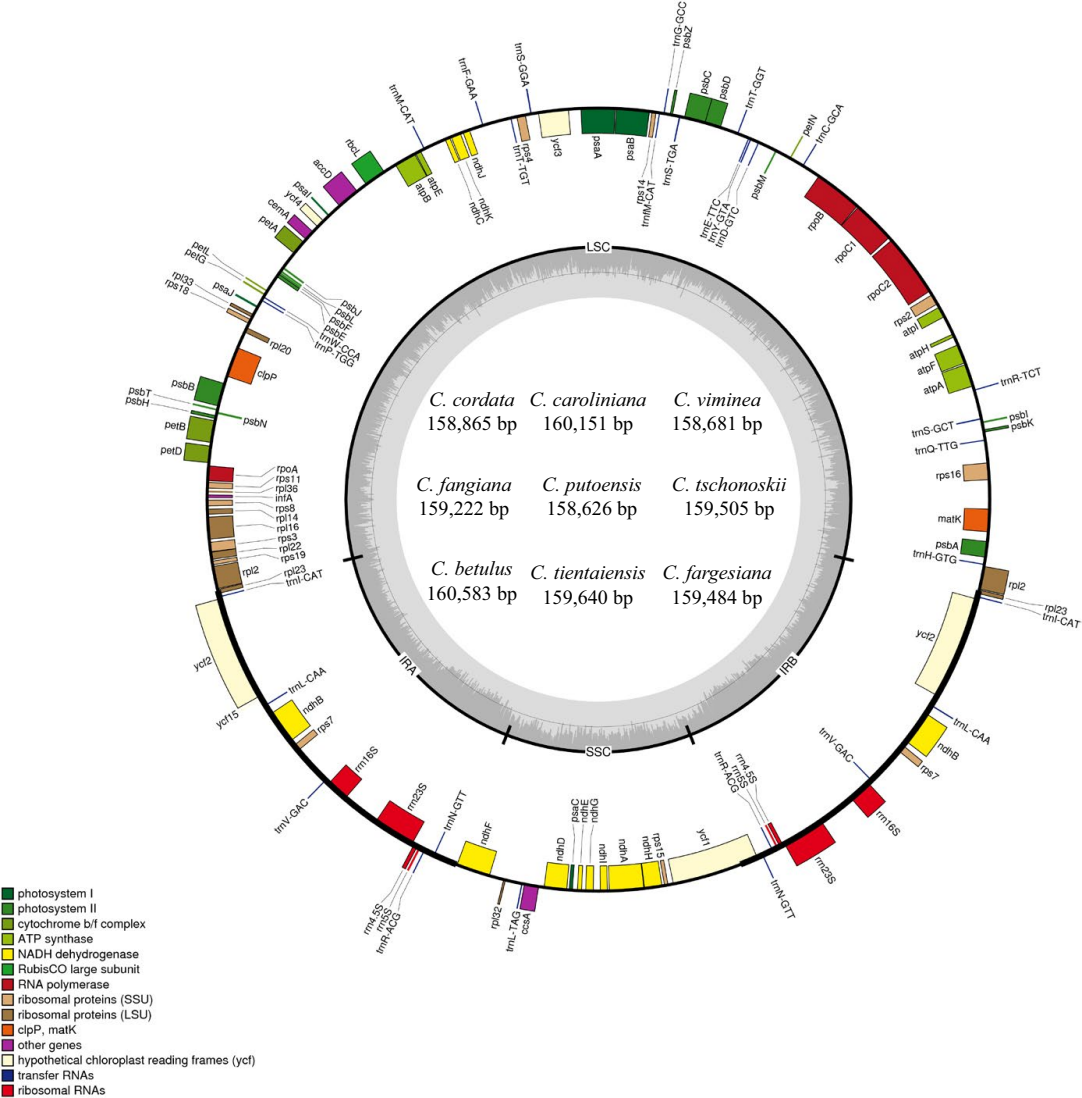
Gene map of the *Carpinus betulus* plastome, as an example of the nine investigated plastomes. Genes drawn outside of the circle are transcribed clockwise, while those inside the circle are transcribed counterclockwise. The typical small single copy (SSC), large single copy (LSC), and inverted repeats (IRa, IRb) are indicated [Colour figure can be viewed at http://wileyonlinelibrary.com]

**Figure 2:**
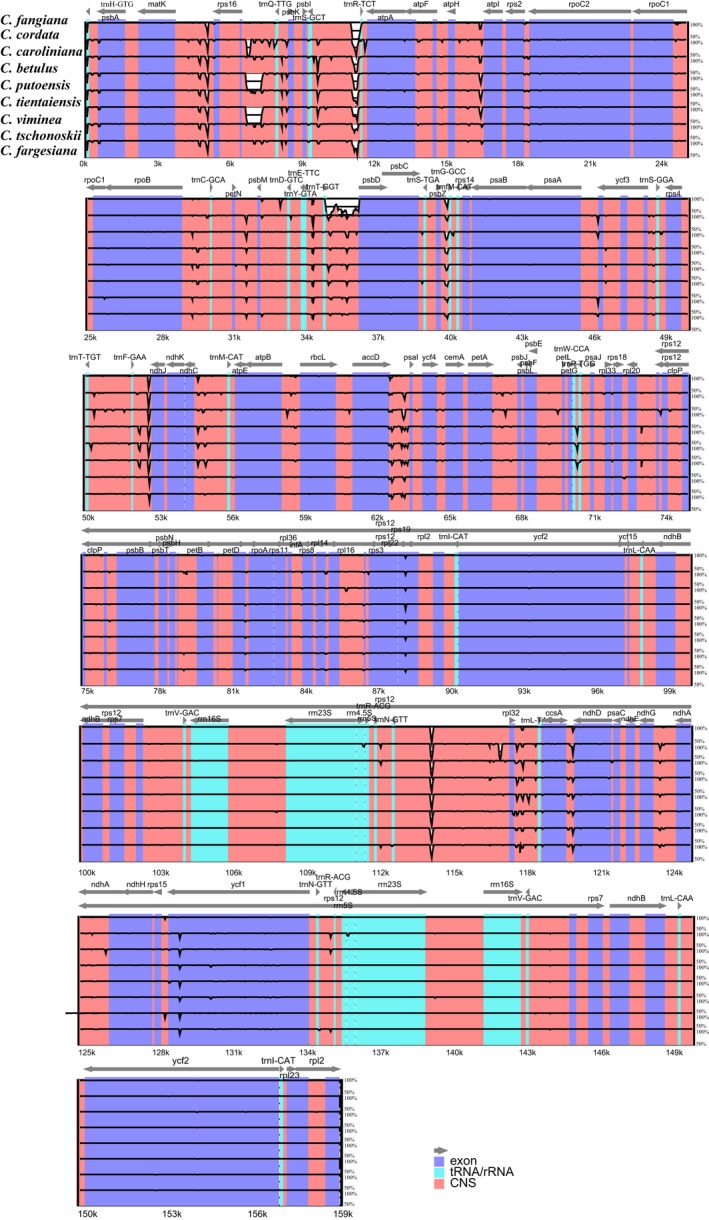
Visualization of alignment of the nine *Carpinus* chloroplast genome sequences. VISTA‐based identity plots showing sequence identity between six sequenced cp genomes of *Carpinus*. Arrows above the alignment indicate genes with their orientation. A cutoff of 70% identity was used for the plots, the Y‐scale indicates the percent identity (50–100%), and the *X*‐axis indicates the coordinate in the chloroplast genome. Exons, rRNA/tRNA, and conserved noncoding sequences (CNS) are marked in purple, blue, and pink, respectively [Colour figure can be viewed at http://wileyonlinelibrary.com]

We found that the nine *Carpinus* plastomes have highly conserved features (Figures 1 & 2), including a typical quadripartite structure consisting of a pair of inverted repeats (IRs; 52,117–55,134 bp) separated by large single copy (LSC; 84,605–84,966 bp) and small single copy (SSC; 17,167–18,825 bp) regions (Table 1). Sizes of the plastomes ranged from 158,626 bp (in *C. putoensis*) to 160,583 bp (in *C. betulus*), and numbers of annotated genes from 121 to 123 (Tables 1, S3). Most genes occurred in single copy, including 75–77 unique protein‐coding genes in the genomes and 18 unique tRNA gene sequences, but there were two copies of all ribosomal RNA genes. Thirteen of the genes were duplicated in the IR regions: four rRNA genes (4.5S, 5S, 16S, and 23S rRNA), four PCGs (*rpl*2*, ycf*2*, ndh*B*, rps*7), and five tRNA genes (*trn*I‐CAT*, trn*L*‐*CAA*, trn*V*‐*GAC*, trn*R*‐*ACG, and *trn*N*‐*GTT). There were also three copies of one gene: *trn*N*‐*GTT. The *rps*12 gene was a unique trans‐spliced gene with three exons. Of the annotated genes, 10 contained a single intron (e.g., *atp*F CDS, *rpo*C1 CDS, and *trn*N‐GTT tRNA), and four protein‐coding genes had two introns (*clp*P, *ycf*3, *rpl*2, and *rps*12). The *rps*19 gene was located in the boundary region between LSC/IRb. Two copies of *ycf*1 gene were located at the junctions of IRb/SSC and SSC/IRa.

**Table 1:** The Chloroplast assembly and annotation information for nine species of *Carpinus*

Species	GenBank	Raw base (G)	Clean base (G)	Entire plastid size (bp)	LSC	SSC	IR (two copies)	Overall GC content (%)	No. of genes	No. of PCGs	No. of rRNA genes	No. of tRNA genes
*C. cordata*	MF977769	3.2	2.9	158865	87938	18800	52127	36.5	122	84	8	29
*C. fangiana*	MF977770	6.1	5.6	159222	88280	18825	52117	36.5	122	84	8	29
*C. betulus*	MF977767	3.2	2.9	160583	88282	17167	55134	36.4	123	85	8	29
*C. caroliniana*	MF977768	2.8	2.5	160151	88555	18513	53083	36.3	123	85	8	29
*C. putoensis*	KX695124	5.0	4.6	158626	87679	18797	52150	36.5	122	84	8	29
*C. tientaiensis*	KY117036	5.6	5.2	159640	88711	18795	52134	36.4	123	85	8	29
*C. viminea*	MF977773	5.4	5.1	158681	87808	18810	52134	36.5	121	83	8	29
*C. tschonoaskii*	MF977772	6.6	6.1	159505	88616	18760	52129	36.4	121	83	8	29
*C. fargesiana*	MF977771	5.8	5.6	159484	88519	18816	52149	36.4	123	85	8	29

Note. LSC: long single copy; SSC: small single copy; PCGs: protein‐coding genes.

In plastomes of each of the nine species, the overall GC content was about 36.5%, and 55% of the plastomes were coding regions (Table 1). All plastomes showed similar features in terms of gene content, gene order, introns, intergenic spacers, and AT content. However, some coding genes were pseudogenized or lost. For example, the *ndh*F gene was lost in *C. cordata, C. fargesiana, C. putoensis*,* C. tientaiensis,* and *C. viminea* and *ndh*I was absent in the latter two species. The intergenic spacers between several pairs of genes varied greatly, for example, between *mat*K & *rps*16, *atp*H & *atp*I, and *trn*S*‐GCT* & *trn*R*‐*TCT (Figure 2).

### 3.2 Phylogenetic analyses

The whole plastome matrix (156,583 bp long) consisted of 1,865 variable sites and 262 parsimony informative sites, while the noncoding dataset (69,308 bp long) included 1,308 variable sites and 190 parsimony informative sites. The coding‐gene dataset (68,058 bp long) comprised only 506 variable sites and 66 parsimony informative sites. The aligned ITS sequence dataset was 623 bp long with 76 variable sites and 53 parsimony informative sites (Table 2).

**Table 2:** Summary of length and variability across different data partitions

Locus	cp genome	LSC	SSC	PCGs	Non‐coding	ITS
Constant sites	154718	84115	16575	67552	68000	547
Parsimony informative sites	262	193	49	66	190	53
Variable sites	1865	1395	321	506	1308	76
Total sites	156583	85510	16896	68058	69308	623
% Parsimony informative sites	0.17	0.23	0.30	0.10	0.27	8.5

Note. LSC: long single copy; SSC: small single copy; PCGs: protein‐coding genes.

**Figure 3:**
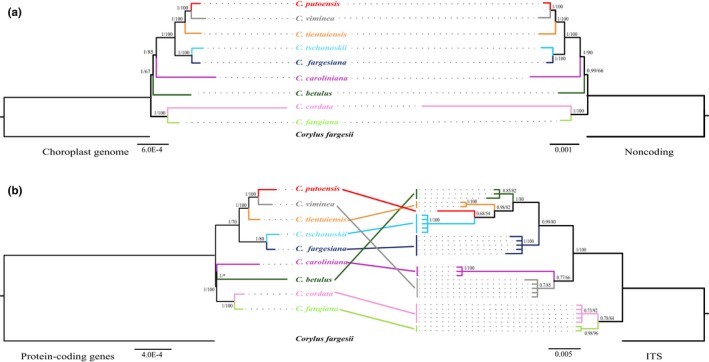
Phylogenetic trees of nine *Carpinus* species based on the three plastome datasets and nrITS sequences, with branch lengths based on results of the Bayesian analyses. Bayesian posterior probabilities (left) and maximum‐likelihood bootstrap values (right) are shown on each node. Different taxa of *Carpinus* and *Corylus* are marked by different colors. (a) Trees based on the chloroplast genome and noncoding regions; (b) trees based on protein coding genes and ITS sequences. *Indicates inconsistent topology based on ML and Bayesian analyses; vertical bars represent different species in the ITS tree [Colour figure can be viewed at http://wileyonlinelibrary.com]

The ML and Bayesian analyses of each chloroplast dataset resulted in similar topologies, but there were discrepancies between those obtained using the plastome and ITS datasets (Figure 3). Phylogenetic analyses of the whole plastome and noncoding datasets (Figure 3a) identified the same topological divergences for all nine species. Five of the seven selected species of section *Carpinus* (*C. tientaiensis*,* C. viminea*,* C. putoensis, C. fargesiana,* and *C. tschonoskii*) and the two species of section *Distegocarpus* (*C. cordata* and *C. fangiana*) formed two well‐supported clades, and topological relationships of these species were the same in all three plastome trees (Figure 3). In the phylogenetic trees derived from analyses of the whole plastomes and noncoding sequences, one of the other species of section *Carpinus* (*C. caroliniana*) was sister to those five species of the section, with high support, while the seventh member (*C. betulus*) was placed sister to the remainder of section *Carpinus* with high posterior probability in the Bayesian analysis, but not the ML analysis (bootstrap values < 70; Figure 3a). The two sections were well separated in these two trees. However, in the trees derived from protein‐coding sequences, *C. caroliniana* and *C. betulus* were grouped together in the ML analysis, but not the Bayesian analysis and clustered with section *Distegocarpus* (Figure 3b), conflicting with the phylogenetic trees based on the other two plastome datasets.

Although the ITS phylogenetic tree also showed high resolution, the topology was mostly incongruent with the phylogenetic trees derived from the plastome datasets (Figure 3b). In the ITS tree, *C. betulus*,* C. tientaiensis*,* C. putoensis,* and *C. tschonoskii* clustered as one clade, while *C. caroliniana* grouped with *C. viminea*, but with low support in both analyses. This pattern of phylogenetic relationships among these seven species is completely incongruent with the patterns in the plastome phylogenetic trees (Figure 3b). Positions of the remaining two species were congruent with the phylogenetic trees based on the whole plastome and noncoding genes datasets, but not the coding genes tree.

## 4 DISCUSSION

Our comparative analyses of plastomes of nine species representing clades identified by Yoo and Wen (2007) suggest that plastid genomes across the genus *Carpinus* are relatively conserved and all have the typical quadripartite structure found in most angiosperm plastomes, including LSC, SSC, and a pair of inverted repeats (IRa and IRb). Total lengths of the nine plastomes range from 158 to 160 kb, and numbers of genes we annotated in them range from 121 to 123. The gene orders and orientations across the nine plastomes are also highly conserved. They all include 75–77 unique protein‐coding genes, 18 unique tRNA gene sequences, and eight ribosomal RNA genes. In addition, the interspecific variations are clearly higher in the noncoding regions, including the intergenic spacers between genes, than in the coding genes (Figure 2), as found in other groups (Hu et al., 2016; Zhang, Ma, & Li, 2011). The conserved and well‐aligned plastomes across different species therefore facilitate the further phylogenetic analyses and comparisons based on the whole plastomes, their coding regions, and noncoding regions.

Previous studies of the genus *Carpinus* or related genera based on a few cpDNA markers have consistently failed to resolve phylogenetic relationships of the major clades (Lu et al., 2016; Yoo & Wen, 2007). In contrast, we obtained well‐supported clades and all interspecific relationships were well resolved except for those of *C. betulus* (Figure 3) by analysis of the whole plastome datasets with more informative sites. It should be noted that we obtained identical topological relationships using the whole plastomes or noncoding datasets. However, results based solely on the coding genes suggested different phylogenetic positions for *C. betulus* and *C. caroliniana* (Figure 3), presumably because the whole plastome and noncoding datasets provided more detailed signals for these two species (Hu et al., 2016; Zeng et al., 2017). These findings suggest that it is essential to assess the consistency of phylogenetic relationships based on whole plastomes and both their coding and noncoding regions, as well as their correspondence to phylogenies derived from analyses of nuclear genes or genomes.

The ITS sequences (623 bp) had a much shorter total length than the coding genes in the plastomes (623 and 68,058 bp, respectively), but included a similar number of parsimony informative sites (53 and 66, respectively). Clearly, the difference in mutation rates implies this may influence estimates of interspecific relationships obtained from analyzing these sets of sequences. The relatively rapid mutation and lineage sorting of the ITS sequence may be helpful for discriminating interspecific relationships for genera such as *Carpinus* (e.g., Lu et al., 2017; Wang, Yu, & Liu, 2011), but in other genera, the ITS sequences may have lower discriminatory power than the chloroplast genes (Hu et al., 2015; Ren et al., 2015). It should be noted that both a single nuclear gene (e.g., ITS) and the plastome (which ultimately represents a single locus) have limited power for resolving a “true” species tree. Multiple, independent nuclear loci or whole genomes would be needed to identify phylogenetic relationships reflecting a “true” species tree, especially when reticulate evolution may have occurred (Hughest, Eastwood, & Bailey, 2006).

The most surprising finding in this study is that the well‐resolved phylogenetic relationships based on plastomes substantially differ from those inferred from the nuclear ITS sequences. Interspecific relationships between all the species except the two members of the basal subclade, *C. cordata* and *C. fangiana*, are inconsistent with those inferred from the three plastome datasets. Such discordance of gene trees derived from nuclear and organelle markers is common and may be due to two nonexclusive factors (Stenz, Larget, Baum, & Ane, 2015; Suh, Smeds, & Ellegren, 2015; Zwickl, Stein, Wing, Ware, & Sanderson, 2014). First, hybridization and introgression are very common in numerous plants (Mallet, 2007), especially wind‐pollinated genera (Abbott, Hegarty, Hiscock, & Brennan, 2010) such as *Carpinus*, in which chloroplast DNA is transmitted solely maternally, via seeds, while nuclear DNA inheritance is bi‐parental, mediated by both pollen and seeds. Long‐distance pollen dispersal and potential hybridizations may have led to the concerted evolution of the ITS sequences towards the introgressing species (Alvarez & Wendel, 2003), while introgressions of the maternally inherited plastomes can only occur when closely related species are geographically close enough (McCauley, Stevens, Peroni, & Raveill, 1996). Both scenarios could distort original phylogenetic relationships of the introgressed species or populations. For example, *C. tschonoskii* and *C. fargesiana* are parapatrically distributed in central China, and introgression events may have caused the observed incongruence between the plastome‐ and ITS‐based trees of the two species. Furthermore, *C. putoensis*, a 14‐ploidy species (Meng, He, Li, & Xu, 2004), is clustered with *C. viminea* in the plastome trees (Figure 3), implying that *C. viminea* or a closely related species was the maternal progenitor during the formation of *C. putoensis*. Its paternal progenitor may be closely related to *C. tschonoskii* according to the interspecific relationships in the ITS tree, but further studies involving more samples and genetic data are needed to better understand the reticulate evolution of *C. putoensis*.

The other factor that could lead to inconsistency between gene trees derived from nuclear and organelle markers is incomplete lineage sorting (ILS) through retention of ancestral polymorphism in different species or populations. This may also lead to inconsistent phylogenies based on different markers with contrasting inheritance (Sousa & Hey, 2013; Suh et al., 2015). When the same ancestral allele is sampled from two distantly related species without complete lineage sorting, the resulting phylogeny will be inconsistent with that based on genes or other DNA sequences following speciation events. The incongruent relationships of *C. betulus* and *C. caroliniana* inferred from the plastome and ITS sequences are probably due to ILS, as these two species occur in Europe and North America, respectively. Thus, they are unlikely to have hybridized with the species in China due to the extreme geographical isolation. Therefore, it is likely that both ILS and hybrid introgression may have been common features of diversifications of the hornbeams. In the future, the genetic evidence from the nuclear genome at the population level will be needed to elucidate the two factors’ precise contributions to the inconsistent phylogenies observed here.

## CONFLICT OF INTEREST

The authors declare no conflict of interests.

## AUTHOR CONTRIBUTIONS

Y.L. and G.R. planned and designed the research. Y.L. carried out the laboratory work and performed the molecular analysis. Y.L. and G.R. wrote the manuscript with the help of Y.Y., Y.L., and X.D.

## DATA ACCESSIBILITY

The GenBank accessions of the whole plastomes of nine species are listed in Table 1 and GenBank accessions of the new generated ITS sequences can be found in Table S2.

## References

[ece34414-bib-0001] Abbott, R. J. , Hegarty, M. J. , Hiscock, S. J. , & Brennan, A. C. (2010). Homoploid hybrid speciation in action. Taxon, 59, 1375–1386.

[ece34414-bib-0002] Alvarez, I. , & Wendel, J. F. (2003). Ribosomal ITS sequences and plant phylogenetic inference. Molecular Phylogenetics and Evolution, 29, 417–434. 14615184 10.1016/s1055-7903(03)00208-2

[ece34414-bib-0003] Bolger, A. M. , Lohse, M. , & Usadel, B. (2014). Trimmomatic: A flexible trimmer for Illumina sequence data. Bioinformatics, 30, 2114–2120. 24695404 10.1093/bioinformatics/btu170 PMC4103590

[ece34414-bib-0004] Cieckiewicz, E. , Angenot, L. , Gras, T. , Kiss, R. , & Frederich, M. (2012). Potential anticancer activity of young *Carpinus betulus* leaves. Planta Medica, 78, 1178. 10.1016/j.phymed.2011.09.072 22014503

[ece34414-bib-0005] van Dijk, E. L. , Jaszczyszyn, Y. , & Thermes, C. (2014). Library preparation methods for next‐generation sequencing: Tone down the bias. Experimental Cell Research, 322, 12–20. 24440557 10.1016/j.yexcr.2014.01.008

[ece34414-bib-0006] Doyle, J. , & Doyle, J. (1987). A rapid DNA isolation procedure for small amounts of fresh leaf tissue. Phytochemical Bulletin, 19, 11–15.

[ece34414-bib-0007] García, N. , Meerow, A. W. , Soltis, D. E. , & Soltis, P. S. (2014). Testing deep reticulate evolution in Amaryllidaceae tribe Hippeastreae (Asparagales) with ITS and chloroplast sequence data. Systematic Botany, 39(1), 75–89.

[ece34414-bib-0009] Hu, H. , Al‐Shehbaz, I. A. , Sun, Y. S. , Hao, G. Q. , Wang, Q. , & Liu, J. Q. (2015). Species delimitation in *Orychophragmus* (Brassicaceae) based on chloroplast and nuclear DNA barcodes. Taxon, 64, 714–726.

[ece34414-bib-0010] Hu, H. , Hu, Q. , Al‐Shehbaz, I. A. , Luo, X. , Zeng, T. , Guo, X. , & Liu, J. (2016). Species delimitation and interspecific relationships of the genus *Orychophragmus* (Brassicaceae) inferred from whole chloroplast genomes. Frontiers in Plant Science, 7, 1826. 27999584 10.3389/fpls.2016.01826 PMC5138468

[ece34414-bib-0011] Huang, D. I. , & Cronk, Q. C. (2015). Plann: A command‐line application for annotating plastome sequences. Applied Plant Sciences, 3, 1–3. 10.3732/apps.1500026 PMC4542940 26312193

[ece34414-bib-0012] Huelsenbeck, J. P. , & Ronquist, F. (2001). MRBAYES: Bayesian inference of phylogenetic trees. Bioinformatics, 17, 754–755. 11524383 10.1093/bioinformatics/17.8.754

[ece34414-bib-0013] Hughest, C. E. , Eastwood, R. J. , & Bailey, C. D. (2006). From famine to feast? Selecting nuclear DNA sequence loci for plant species‐level phylogeny reconstruction. Philosophical Transactions of the Royal Society of London. Series B, Biological Sciences, 361, 211–225. 16553318 10.1098/rstb.2005.1735 PMC1626539

[ece34414-bib-0014] Jansen, R. K. , Cai, Z. , Raubeson, L. A. , Daniell, H. , Depamphilis, C. W. , Leebens‐Mack, J. , … Boore, J. L. (2007). Analysis of 81 genes from 64 plastid genomes resolves relationships in angiosperms and identifies genome‐scale evolutionary patterns. Proceedings of the National Academy of Sciences of the United States of America, 104, 19369–19374. 18048330 10.1073/pnas.0709121104 PMC2148296

[ece34414-bib-0015] Katoh, K. , Misawa, K. , Kuma, K , & Miyata, T. (2002). MAFFT: A novel method for rapid multiple sequence alignment based on fast Fourier transform. Nucleic Acids Research, 30, 3059–3066. 12136088 10.1093/nar/gkf436 PMC135756

[ece34414-bib-0016] Kearse, M. , Moir, R. , Wilson, A. , Stones‐Havas, S. , Cheung, M. , Sturrock, S. , … Drummond, A. (2012). Geneious Basic: An integrated and extendable desktop software platform for the organization and analysis of sequence data. Bioinformatics, 28, 1647–1649. 22543367 10.1093/bioinformatics/bts199 PMC3371832

[ece34414-bib-0333] Kuang, K.‐Z. , & Li, P.‐C. (1979). FRPS. Flora Reipublicae Popularis Sinicae. Myricaeae, Juglandaceae, Betulaceae (pp. 58–89). Beijing, China: Science Press 21.

[ece34414-bib-0017] Langmead, B. , & Salzberg, S. L. (2012). Fast gapped‐read alignment with Bowtie 2. Nature Methods, 9, 357–359. 22388286 10.1038/nmeth.1923 PMC3322381

[ece34414-bib-0018] Larkin, M. A. , Blackshields, G. , Brown, N. P. , Chenna, R. , McGettigan, P. A. , McWilliam, H. , … Higgins, D. G. (2007). Clustal W and clustal X version 2.0. Bioinformatics, 23, 2947–2948. 17846036 10.1093/bioinformatics/btm404

[ece34414-bib-0019] Li, Y. , Bi, H. , Liu, B. , Guo, X. , Hao, G. , He, Q. , & Ma, T. (2016). The complete chloroplast genome of *Ostrya rehderiana* . Mitochondrial DNA Part A, 27, 4536–4537. 10.3109/19401736.2015.1101551 26540005

[ece34414-bib-0020] Li, H. , & Durbin, R. (2009). Fast and accurate short read alignment with Burrows–Wheeler transform. Bioinformatics, 25, 1754–1760. 19451168 10.1093/bioinformatics/btp324 PMC2705234

[ece34414-bib-0021] Li, H. , Handsaker, B. , Wysoker, A. , Fennell, T. , Ruan, J. , Homer, N. , … Genome Project Data Processing S (2009). The sequence alignment/map format and SAMtools. Bioinformatics, 25, 2078–2079. 19505943 10.1093/bioinformatics/btp352 PMC2723002

[ece34414-bib-0022] Li, P. , & Skvortsov, A. (1999). Betulaceae. Science Press, 4, 289–300.

[ece34414-bib-0220] Lindenbaum, P. (2015). JVarkit: java‐based utilities for Bioinformatics. Figshare. Retrieved from: http://dxdoiorg/106084/m9figshare1425030.

[ece34414-bib-0222] Lohse, M. , Drechsel, O. , Kahlau, S. , & Bock, R. (2013). OrganellarGenomeDRAW—a suite of tools for generating physical maps of plastid and mitochondrial genomes and visualizing expression data sets. Nucleic Acids Research, 41, 75–81. 10.1093/nar/gkt289 PMC3692101 23609545

[ece34414-bib-0024] Loytynoja, A. , & Goldman, N. (2010). webPRANK: A phylogeny‐aware multiple sequence aligner with interactive alignment browser. BMC Bioinformatics, 11, 579. 21110866 10.1186/1471-2105-11-579 PMC3009689

[ece34414-bib-0025] Lu, Z. Q. , Liu, S. Y. , Yang, X. Y. , Liang, Q. L. , Yang, Y. Z. , Zhang, D. , … Liu, J. Q. (2017). *Carpinus langaoensis* (Betulaceae), a new hornbeam species from the Daba Mountains in Shaanxi, China. Phytotaxa, 295, 185–193.

[ece34414-bib-0026] Lu, Z. Q. , Zhang, D. , Liu, S. Y. , Yang, X. Y. , Liu, X. , & Liu, J. Q. (2016). Species delimitation of Chinese hop‐hornbeams based on molecular and morphological evidence. Ecology and Evolution, 6, 4731–4740. 27547308 10.1002/ece3.2251 PMC4979702

[ece34414-bib-0027] Mallet, J. (2007). Hybrid speciation. Nature, 446, 279–283. 17361174 10.1038/nature05706

[ece34414-bib-0028] Mallet, J. , Besansky, N. , & Hahn, M. W. (2016). How reticulated are species? BioEssays, 38, 140–149. 26709836 10.1002/bies.201500149 PMC4813508

[ece34414-bib-0029] Mayor, C. , Brudno, M. , Schwartz, J. R. , Poliakov, A. , Rubin, E. M. , Frazer, K. A. , … Dubchak, I. (2000). VISTA: Visualizing global DNA sequence alignments of arbitrary length. Bioinformatics, 16, 1046–1047. 11159318 10.1093/bioinformatics/16.11.1046

[ece34414-bib-0030] McCauley, D. E. , Stevens, J. E. , Peroni, P. A. , & Raveill, J. A. (1996). The spatial distribution of chloroplast DNA and allozyme polymorphisms within a population of *Silene alba* (Caryophyllaceae). American Journal of Botany, 83, 727–731.

[ece34414-bib-0031] Meng, A. , He, Z. , Li, J. , & Xu, L. (2004). Chromosome numbers of two threatened species of Betulaceae. Wuhan Botanical Research, 22, 171–173.

[ece34414-bib-0032] Rambaut, A. S. M. , Xie, D. , & Drummond, A. (2014). Tracer v. 1.6. Institute of Evolutionary Biology University of Edinburgh. Retrieved from: http://beast.bio.ed.ac.uk/Tracer.

[ece34414-bib-0033] Ren, G. , Conti, E. , & Salamin, N. (2015). Phylogeny and biogeography of *Primula* sect. *Armerina*: Implications for plant evolution under climate change and the uplift of the Qinghai‐Tibet Plateau. BMC Evolutionary Biology, 15, 161. 26275399 10.1186/s12862-015-0445-7 PMC4537560

[ece34414-bib-0035] Sheng, Q. Q. , Fang, X. Y. , Zhu, Z. L. , Xiao, W. , Wang, Z. Z. , Ding, G. , … Sun, Q. (2016). Seasonal variation of pheophorbide a and flavonoid in different organs of two *Carpinus* species and its correlation with immunosuppressive activity. In Vitro Cellular & Developmental Biology – Animal, 52, 654–661. 27112162 10.1007/s11626-016-0041-1

[ece34414-bib-0036] Sousa, V. , & Hey, J. (2013). Understanding the origin of species with genome‐scale data: Modelling gene flow. Nature Reviews Genetics, 14, 404–414. 10.1038/nrg3446 PMC5568773 23657479

[ece34414-bib-0037] Stamatakis, A. (2014). RAxML version 8: A tool for phylogenetic analysis and post‐analysis of large phylogenies. Bioinformatics, 30, 1312–1313. 24451623 10.1093/bioinformatics/btu033 PMC3998144

[ece34414-bib-0038] Stenz, N. W. M. , Larget, B. , Baum, D. A. , & Ane, C. (2015). Exploring tree‐like and non‐tree‐like patterns using genome sequences: An example using the inbreeding plant species *Arabidopsis thaliana* (L.) Heynh. Systematic Biology, 64, 809–823. 26117705 10.1093/sysbio/syv039

[ece34414-bib-0039] Suh, A. , Smeds, L. , & Ellegren, H. (2015). The dynamics of incomplete lineage sorting across the ancient adaptive radiation of Neoavian birds. Plos Biology, 13, e1002224. 26284513 10.1371/journal.pbio.1002224 PMC4540587

[ece34414-bib-0040] Tamura, K. , Stecher, G. , Peterson, D. , Filipski, A. , & Kumar, S. (2013). MEGA6: Molecular evolutionary genetics analysis version 6.0. Molecular Biology and Evolution, 30, 2725–2729. 24132122 10.1093/molbev/mst197 PMC3840312

[ece34414-bib-0041] Wang, Q. , Yu, Q. S. , & Liu, J. Q. (2011). Are nuclear loci ideal for barcoding plants? A case study of genetic delimitation of two sister species using multiple loci and multiple intraspecific individuals. Journal of Systematics and Evolution, 49, 182–188.

[ece34414-bib-0042] Xu, B. , Wu, N. , Gao, X. F. , & Zhang, L. B. (2012). Analysis of DNA sequences of six chloroplast and nuclear genes suggests incongruence, introgression, and incomplete lineage sorting in the evolution of *Lespedeza* (Fabaceae). Molecular Phylogenetics and Evolution, 62, 346–358. 22032991 10.1016/j.ympev.2011.10.007

[ece34414-bib-0043] Yoo, K. O. , & Wen, J. (2007). Phylogeny of *Carpinus* and subfamily *Coryloideae* (Betulaceae) based on chloroplast and nuclear ribosomal sequence data. Plant Systematics and Evolution, 267, 25–35.

[ece34414-bib-0044] Zeng, S. Y. , Zhou, T. , Han, K. , Yang, Y. C. , Zhao, J. H. , & Liu, Z. L. (2017). The complete chloroplast genome sequences of six *Rehmannia* species. Genes‐Basel, 8, 103. 10.3390/genes8030103 PMC5368707 28294981

[ece34414-bib-0045] Zerbino, D. R. , & Birney, E. (2008). Velvet: Algorithms for de novo short read assembly using de Bruijn graphs. Genome Research, 18, 821–829. 18349386 10.1101/gr.074492.107 PMC2336801

[ece34414-bib-0046] Zhang, Y. ‐J. , & Li, D. ‐Z. (2011). Advances in phylogenomics based on complete chloroplast genomes. Plant Diversity & Resources, 6, 365–375.

[ece34414-bib-0047] Zhang, Y. J. , Ma, P. F. , & Li, D. Z. (2011). High‐throughput sequencing of six bamboo chloroplast genomes: Phylogenetic implications for temperate woody bamboos (Poaceae: Bambusoideae). PLoS ONE, 6, e20596. 21655229 10.1371/journal.pone.0020596 PMC3105084

[ece34414-bib-0048] Zwickl, D. J. , Stein, J. C. , Wing, R. A. , Ware, D. , & Sanderson, M. J. (2014). Disentangling methodological and biological sources of gene tree discordance on *Oryza* (Poaceae) chromosome 3. Systematic Biology, 63, 645–659. 24721692 10.1093/sysbio/syu027

