# Supplementary material for: Plastomes of nine hornbeams and phylogenetic implications
Source: Ecol Evol. 2018 Aug 7;8(17):8770–8. doi: 10.1002/ece3.4414 (PMC6157693; doi:10.1002/ece3.4414)
Supplement: Supplementary file 1 [file ECE3-8-8770-s001.docx]

**Table S1** Collection locations for tissue samples used to generate plastome sequences of nine *Carpinus* species.

| **Species** | **Location** | **Latitude** | **Longitude** | **Altitude** |
| --- | --- | --- | --- | --- |
|  |  | **(N)** | **(E)** | **(m)** |
| *C. cordata* | Langao, Shanxi | 32.128° | 108.804° | 1223 |
| *C. fangiana* | Ebian, Sichuan | 29.040° | 103.008° | 1671 |
| *C. betulus* | Caucasus, Dagestan | - | - | - |
| *C. caroliniana* | Savanna Portage State Park, USA | 40.837° | 93.157° | 390 |
| *C. putoensis* | Zhoushan, Zhejiang | 30.009° | 122.392° | 280 |
| *C. tientaiensis* | Taiantai, Zhejiang | 29.170° | 121.048° | 860 |
| *C. viminea* | Huoshan, Anhui | 30.982° | 116.079° | 1080 |
| *C. tschonskii* | Nanjiang, Sichuan | 32.621° | 106.830° | 1710 |
| *C. fargesiana* | Lushi, Henan | 33.745° | 106.830° | 1710 |

“-”: null;
