# Supplementary material for: Plastomes of nine hornbeams and phylogenetic implications
Source: Ecol Evol. 2018 Aug 7;8(17):8770–8. doi: 10.1002/ece3.4414 (PMC6157693; doi:10.1002/ece3.4414)
Supplement: Supplementary file 2 [file ECE3-8-8770-s002.docx]

**TABLE S2** GenBank accessions for ITS sequences of nine *Carpinus* species used in this study.

| **Species** | **Genbank number** |
| --- | --- |
|  |  |
| *C. cordata* | MG569952 |
| *C. cordata* | MG569953 |
| *C. cordata* | MG569954 |
| *C. cordata* | MG569955 |
| *C. cordata* | MG569956 |
| *C. fangiana* | MG569957 |
| *C. fangiana* | MG569958 |
| *C. betulus* | AF297362 |
| *C. betulus* | AF297362 |
| *C. betulus* | AF297362 |
| *C. caroliniana* | AJ783634 |
| *C. caroliniana* | AJ783634 |
| *C. caroliniana* | AJ783634 |
| *C. putoensis* | KX946972 |
| *C. tientaiensis* | KX946975 |
| *C. tientaiensis* | JF796534 |
| *C. viminea* | MG569969 |
| *C. viminea* | MG569970 |
| *C. viminea* | MG569971 |
| *C. viminea* | MG569972 |
| *C. viminea* | MG569973 |
| *C. tschonskii* | MG569964 |
| *C. tschonskii* | MG569965 |
| *C. tschonskii* | MG569966 |
| *C. tschonskii* | MG569967 |
| *C. tschonskii* | MG569968 |
| *C. fargesiana* | MG569959 |
| *C. fargesiana* | MG569960 |
| *C. fargesiana* | MG569961 |
| *C. fargesiana* | MG569962 |
| *C. fargesiana* | MG569963 |
