# Supplementary material for: Plastomes of nine hornbeams and phylogenetic implications
Source: Ecol Evol. 2018 Aug 7;8(17):8770–8. doi: 10.1002/ece3.4414 (PMC6157693; doi:10.1002/ece3.4414)
Supplement: Supplementary file 3 [file ECE3-8-8770-s003.docx]

| **Category for genes** | **Group of genes** | **Name of genes** | | | | |  |  |
| --- | --- | --- | --- | --- | --- | --- | --- | --- |
| **Self replication** | Ribosomal RNA genes | rrn16^b^ | rrn23^b^ | rrn4.5^b^ | rrn5^b^ |  |  |  |
|  | Transfer RNA genes | trnH-GTG | trnQ-TTG | trnS-GCT | trnR-TCT | trnC-GCA |  |  |
|  |  | trnD-GTC | trnY-GTA | trnE-TTC | trnT-GGT | trnS-TGA |  |  |
|  |  | trnG-GCC | trnfM-CAT | trnS-GGA | trnT-TGT | trnF-GAA |  |  |
|  |  | trnM-CAT | trnW-CCA | trnP-TGG | trnI-CAT^b^ | trnL-CAA^b^ |  |  |
|  |  | trnV-GAC^b^ | trnR-ACG^b^ | trnN-GTT^a,c^ | trnL-TAG |  |  |  |
|  | Small subunit of ribosome | rps12^a^ | rps16^a^ | rps2 | rps14 | rps4 | rps18 |  |
|  |  | rps11 | rps8 | rps3 | rps19 | rps7^b^ | rps15 |  |
|  | Large subunit of ribosome | rpl33 | rpl20 | rpl36 | rpl14 | rpl16^a^ | rpl22 | rpl2^a,b^ |
|  |  | rpl23 | rpl32 |  |  |  |  |  |
|  | DNA dependent RNA polymerase | rpoC2 | rpoC1^a^ | rpoB | rpoA |  |  |  |
|  | Translational initiation factor | InfA |  |  |  |  |  |  |
| **Genes for photosynthesis** | Subunits of photosystem I | psaB | psaA | psaI | psaJ | psaC |  |  |
|  | Subunits of photosystem II | psbA | psbK | psbI | psbM | psbD | psbC | psbZ |
|  |  | psbL | psbF | psbE | psbB | psbT | psbN | psbH |
|  |  | psbJ |  |  |  |  |  |  |
|  | Subunits of cytochrome | petN | petA | petL | petG | petB^a^ | petD^a^ |  |
|  | Subunits of ATP synthase | AtpA | atpF^a^ | AtpH | AtpI | atpE | atpB |  |
|  | ATP-dependent protease subunit p gene | clpP^a^ |  |  |  |  |  |  |
|  | Large subunit of Rubisco | rbcL |  |  |  |  |  |  |
|  | Subunits of NADH dehydrogenase | ndhJ | ndhK | ndhC | ndhB^a,b^ | ndhF^d,e^ | ndhD | ndhE |
|  |  | ndhG | ndhI^e^ | ndhA^a^ | NdhH |  |  |  |
| **Other genes** | Maturase | matK |  |  |  |  |  |  |
|  | Envelop membrane protein | cemA |  |  |  |  |  |  |
|  | Subunit of acetyl-CoA-carboxylase | accD |  |  |  |  |  |  |
|  | c-type cytochrome synthesis gene | ccsA |  |  |  |  |  |  |
| **Genes of unknown function** | Conserved open reading frames | ycf3^a^ | ycf4 | ycf2^b^ | ycf15 | ycf1 |  |  |

**TABLE S3** The gene constitution in the chloroplast genome of nine species of Carpinus used in this study.

^a^ indicates the existence of intron in the corresponding genes; ^b^ indicates the corresponding gene located in IR region; ^c^ indicates the gene have two copy. ^d^ indicates *C. crdata, C. fargesiana* and *C. putoensis* lacking the corresponding genes; ^e^ indicates *C. tientaiensis* and *C. viminea* lacking the corresponding genes.
